# Deoxy-Piezo1 hyperactivity elevates pump-leak fluxes and lactate production in sickle cells

**DOI:** 10.1016/j.bpj.2026.04.008

**Published:** 2026-04-10

**Authors:** Virgilio L. Lew, Simon D. Rogers

**Affiliations:** 1 Physiological Laboratory, Department of Physiology, Development and Neuroscience, University of Cambridge, Downing Street, Cambridge CB2 3EG, UK; 2 School of Computing Science, University of Glasgow, S123, Sir Alwyn Williams Building, Glasgow G12 8RZ, UK

## Abstract

Sickle cell disease, a highly debilitating disease affecting millions worldwide, is caused by the homozygous inheritance of the mutant hemoglobin S (HbS), a malaria-stabilized gene conferring protection against lethal cerebral malaria. When sickle red blood cells traverse deoxygenated bloodstreams, HbS rapidly nucleates into polymers that keep the sickle cell Piezo1 channels open for the duration of deoxy transits. On transition to oxy streams, Piezo1 channels close immediately and remain closed. Downgradient ion fluxes (“leaks”) through open deoxy-Piezo1 channels change the intracellular concentrations of Ca^2+^, K^+^, and Na^+^ in sickle cells stimulating balancing counter-fluxes via the calcium (PMCA) and Na/K (ATP1) membrane pumps. This sets up a complex Ca^2+^, Na^+^, and K^+^ pump-leak flux-dynamics in the sickle cells. The current investigation is focused on the magnitude, kinetics, and implications of the pump-leak fluxes induced by deoxy-Piezo1 channels on the pathophysiology of sickle cell disease. Model simulations were used to compare and contrast the pump-leak calcium flux patterns of normal and sickle red blood cells. The results predicted a unique kinetic pattern for the pump-leak calcium fluxes in deoxy sickle cells: a sharp calcium influx peak on capillary ingress followed by a near-zero net calcium flux for the duration of deoxy transits, masking large unidirectional and energy consuming pump-leak calcium fluxes. The same pattern applied to pump-leak Na^+^ and K^+^ fluxes, with particular intensity in the irreversibly sickled cells. Estimates of ATP turnover and lactate production rates associated with pump-leak Ca^2+^, Na^+^, and K^+^ fluxes were found to be substantially increased above those in normal, oxy, or deoxy RBCs or in oxy sickle RBCs. These results opened the possibility that pump-mediated increased ATP turnover in deoxy sickle cells contributes significantly to lactate production and metabolic acidosis in sickle cell disease.

## Significance

Experimental and modeling studies on sickle cells revealed a major role of hyperactive deoxy-Piezo1 channels on the pathophysiology of sickle cell disease. Those results suggested a possible origin for the unknown acid source causing metabolic acidosis in sickle cell anemia patients, a condition often associated with clinical severity and pain crises. Acidosis results from increased lactate production, impaired metabolism by liver enzymes, and reduced excretion by the kidney, organs variably compromised in sickle cell disease. The drastically elevated pump-leak fluxes documented here in deoxygenated sickle cells increase ATP turnover and metabolic lactate production. A detailed analysis of the model predictions suggests that sickle cell pump-leak activity could be the main source of lactic acid in metabolic acidotic patients.

## Introduction

Red blood cells (RBCs) from healthy human adults (normal RBCs) show remarkable stability for long circulatory lifespans of about 4 months under relentless hemodynamic motion and homeostatic challenges caused by myriad capillary crossings and gas exchanges. Longevity minimizes the energy required for RBC biosynthetic renewal by reducing its frequency, a critical energy saving considering that RBCs constitute the largest cell mass of the human body. In addition, the constitutional low permeability of the RBC membrane to sodium, potassium, calcium, and magnesium, one of the lowest in nature, also minimizes the metabolic costs of sustaining balanced ionic traffic, stable volume, and composition in the face of continuous oxy-deoxy transitions and hemodynamic changes, a state often described as in overall pump-leak balance.^1^^,^^2^ “Pump-leak” will be used here in a narrower sense, as a shorthand to describe the circulatory dynamics of the net fluxes of Ca^2+^, Na^+^, and K^+^ through the calcium and Na/K pumps of the RBC plasma membrane (the “pump”) and the downgradient passive fluxes of these ions through Piezo1 and Gardos channels (the “leak”).

Capillary crossings pose the most relentless functional challenge to RBCs. RBC deformation on capillary entry activates a fraction of the Piezo1 channels in the RBC membrane for a few milliseconds, transiently increasing the plasma membrane ionic permeability.^3^^,^^4^^,^^5^ Consecutive capillary transits between oxy-deoxy bloodstreams allow rapid CO_2_-O_2_ exchanges between RBCs and tissue cells associated with sharp and reversible changes in the isoelectric point of hemoglobin.^6^^,^^7^^,^^8^^,^^9^^,^^10^ In turn, the oxy-deoxy changes in the isoelectric point affect cell pH and the membrane potential, causing secondary effects on many ion transport and metabolic processes.^11^ After ∼200,000 capillary transits, aging normal RBCs show hardly any decay in their gas transport efficiency and rheological fitness.^12^ This level of functional optimization is achieved with minimal metabolic cost, less than 0.02% of the normal daily ATP turnover of an adult organism.^1^

Longevity and functional optimizations are variably disrupted in many genetic diseases affecting RBCs. The focus of the current study is on sickle cell anemia RBCs. When a sickle cell enters deoxy bloodstreams a random fraction of its Piezo1 channels open each time and remain open for the duration of the deoxy transit.^13^^,^^14^^,^^15^ This exposes the cell to long periods of elevated ion permeability in the circulation, vastly increasing downgradient calcium, sodium, and potassium fluxes relative to normal RBCs. The resulting intracellular concentration changes elicit, in turn, compensatory calcium and sodium extrusion and potassium gain fluxes via ATP-consuming calcium (PMCA) and Na/K pumps (ATP1), thus increasing the energy consumption, metabolic ATP turnover, and lactate production of sickle cells.

For an understanding of the mechanisms behind this dynamic balance, and of its effects on the RBCs and on the organism as a whole, it is necessary to compare the magnitudes and kinetic patterns of the pump-leak fluxes over single and multiple capillary crossings, a challenge not yet accessible experimentally but amenable to study with a modeling approach. The large body of experimental and clinical results on sickle cells accumulated over the last century provides a solid body of verified facts allowing model parameters of RBC homeostasis to be tightly constrained.^14^^,^^15^

Besides reticulocytes and macrocytic stress reticulocytes, the three dominant sickle cell subtypes found in variable proportions in blood samples of sickle cell anemia patients are diskocytes, carrying 100% HbS; F-cells, carrying a variable mix of HbS and fetal hemoglobin, HbF; and irreversibly sickled cells (ISCs), also carrying 100% HbS.^16^^,^^17^^,^^18^ The circulatory lifespans of these main subtypes differ markedly, about 2 weeks for diskocytes, 6–8 weeks for F-cells,^19^ and 4–7 days for ISCs.^17^^,^^20^^,^^21^

ISCs play a major role in the pathophysiology of sickle cell disease as main participants in vaso-occlusion, the root cause of organ failure and pain crisis in this disease.^22^^,^^23^^,^^24^^,^^25^ Unlike diskocytes and F-cells that mature more or less normally from reticulocytes within about 3 days after bone marrow release, ISCs originate from a separate, 100% HbS stress-reticulocyte macrocytic lineage^14^^,^^15^^,^^26^^,^^27^ and remain in an arrested maturation state in the circulation, with reduced HbS contents, organelle retention, very high transport and metabolic activity, and vast calcium-accumulating capacity within internal vesicles.^28^^,^^29^ ISCs dehydrate maximally within a day or so after bone marrow release^17^ and remain in a low-potassium, high-sodium hyperdense state, the pathogenic state, for most of their lifespan.^15^

Romero et al.^30^ recently applied an original methodological design that allowed, for the first time, the separation of ISCs from all other sickle cell subtypes in sufficient numbers to perform comparative studies, statistical analyses, and direct measurements of Piezo1-mediated currents in inside-out membrane patches from human and mice sickle cells. Their results showed that Piezo1-mediated currents in ISCs were substantially elevated relative to those in the non-ISC cells, even in oxy conditions, and that the channel kinetics in response to mechano-pressure pulses in ISCs differed from that in non-ISCs. In mice sickle cells, a diet enriched in the ω-3 acid eicosapentaenoic fatty acid normalized PIEZO1 activity and reduced hypoxic ISC formation, supporting the view that the upregulated constitutive condition of Piezo1 in developmentally arrested ISCs was linked to the immature lipid composition of the membrane.^14^^,^^15^^,^^31^^,^^32^

The three main sickle cell subtypes dehydrate and densify by the same well-known [Ca^2+^]_i_-dependent mechanism mediated by Gardos channels.^14^ Dehydration is driven by the outward potassium gradient, leading to KCl and water loss. In vivo, dehydration rates differ markedly between sickle cell subtypes with ISCs by far the fastest. Normal and sickle RBCs share a common terminal density reversal process resulting from late reduction in Na/K pump activity, by pump decay in normal RBCs, diskocytes, and F-cells, and by metabolic exhaustion in ISCs.^11^^,^^15^^,^^33^ With weakened Na/K pumps, aging RBCs are no longer able to balance the Piezo1-mediated sodium and potassium fluxes, gain NaCl in excess of KCl losses, and rehydrate terminally.

The current study attempts to elucidate the circulatory pump-leak dynamic triggered by enhanced deoxy-Piezo1 activity in the different sickle cell subtypes and to explore the potential link between increased pump-mediated ATP turnover and metabolic acidosis, essential knowledge for understanding the mechanisms operating in vivo behind the pathophysiology of sickle cell disease.

## Materials and methods

The aim of this model study was to estimate the extent to which the increased permeability of sickle cells in the deoxy circulation stimulates pump-mediated calcium and sodium fluxes, ATP consumption, and lactate generation in vivo. The permeability increase was attributed in the model to the condition of Piezo1 channels in deoxygenated sickle cells. This attribution was based on a vast amount of verified experimental evidence accumulated over decades in different laboratories worldwide, much of it under the name of Psickle.^18^^,^^34^ The long assumed identity of Piezo1 as Psickle has recently been firmly confirmed experimentally by the results of Romero et al.^30^ validating model predictions advanced in previous studies in sickle cells.^14^^,^^15^

**Figure 1:**
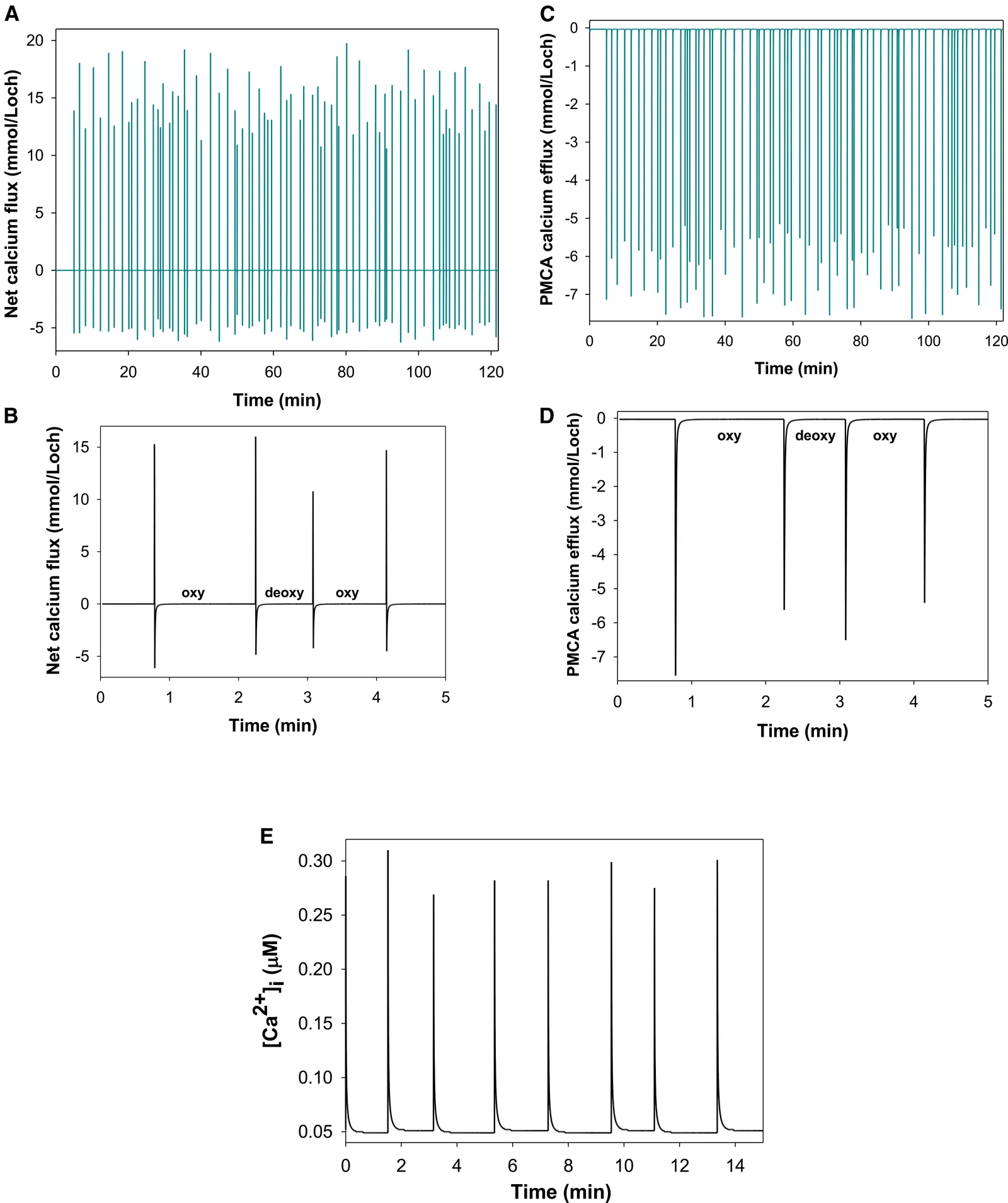
Patterns of Piezo1-induced calcium fluxes in normal red blood cells during capillary transits. (A) The subsecond opening of Piezo1 channels on capillary entries triggers a net downgradient calcium influx shown as positive upward peaks, immediately followed by negative troughs caused by PMCA-mediated extrusion of residual cell calcium after Piezo1 closure. The near-zero net flux line between peak-trough pairs shows that pump-leak calcium fluxes between capillary transits remain balanced at baseline physiological pump-leak levels.^37^ (B) Same as (A) on an expanded timescale showing identical peak-trough patterns for oxy-deoxy and deoxy-oxy transitions. (C) PMCA-mediated calcium extrusion fluxes always rapidly returning to baseline levels between capillary transits. (D) Same as (C) on an expanded timescale to show the high speed at which the PMCA returns to baseline flux levels in between transits regardless of oxy or deoxy transitions. (E) Piezo1-induced [Ca^2+^]_i_ changes during capillary crossing, from baseline levels around 50 nM to peak levels four- to sixfold higher for a few seconds after each crossing.

**Figure 3:**
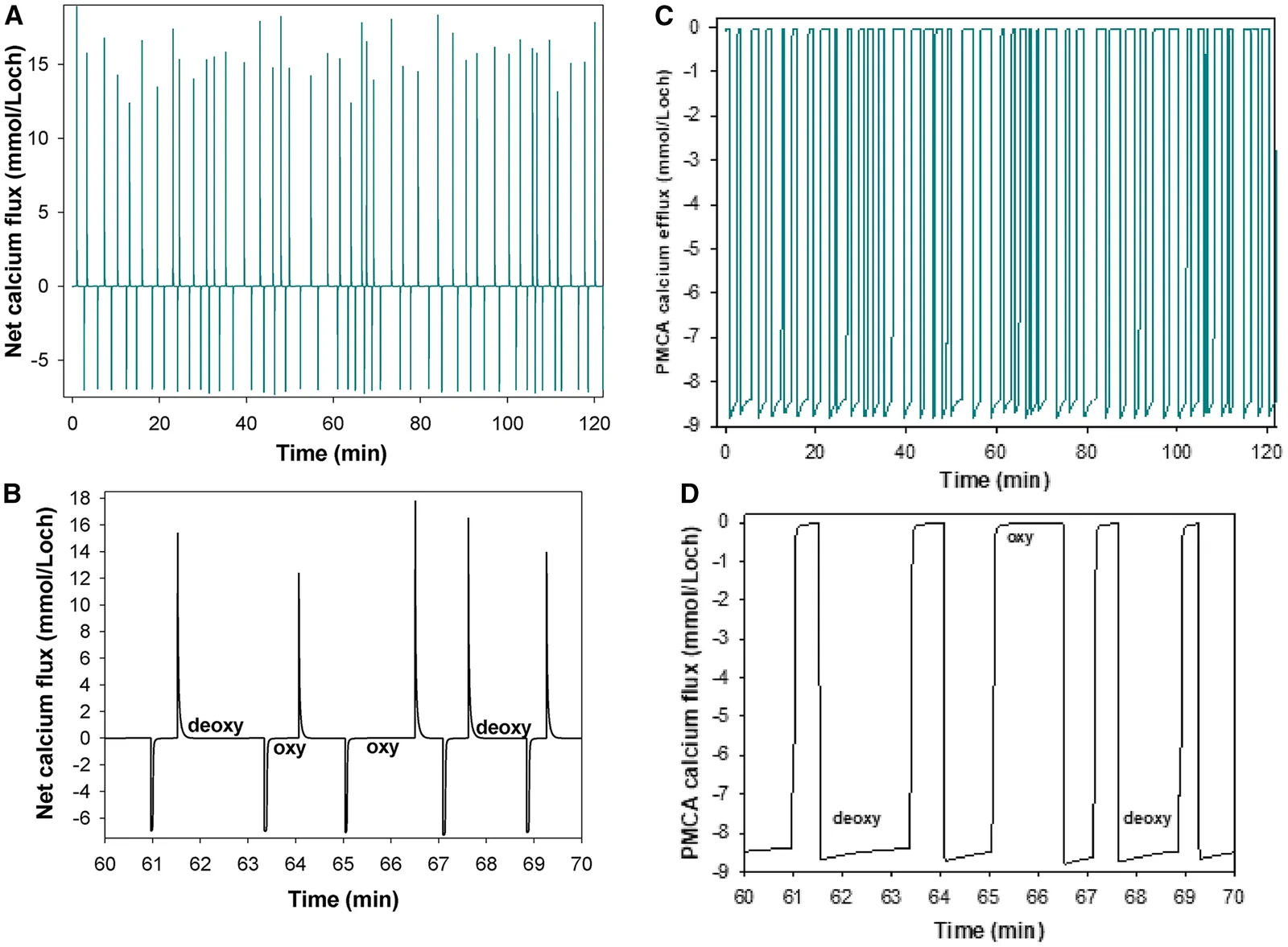
Patterns of Piezo1-induced calcium fluxes in sickle diskocytes during capillary transits (A–D) report the same variables as (A)–(D) of Figures 1, 3, and 5 for optimal comparability. The parameters defining the Piezo1-mediated permeabilities and maximal PMCA-mediated calcium extrusion rates were set in the simulation protocols within similar ranges for both normal RBCs and sickle diskocytes. In this way sickle diskocytes responses differing from those of normal RBCs and sickle F-cells could only be attributed to deoxy-Piezo1 channels remaining open in the sickle diskocytes for the full duration of deoxy transits.

**Figure 4:**
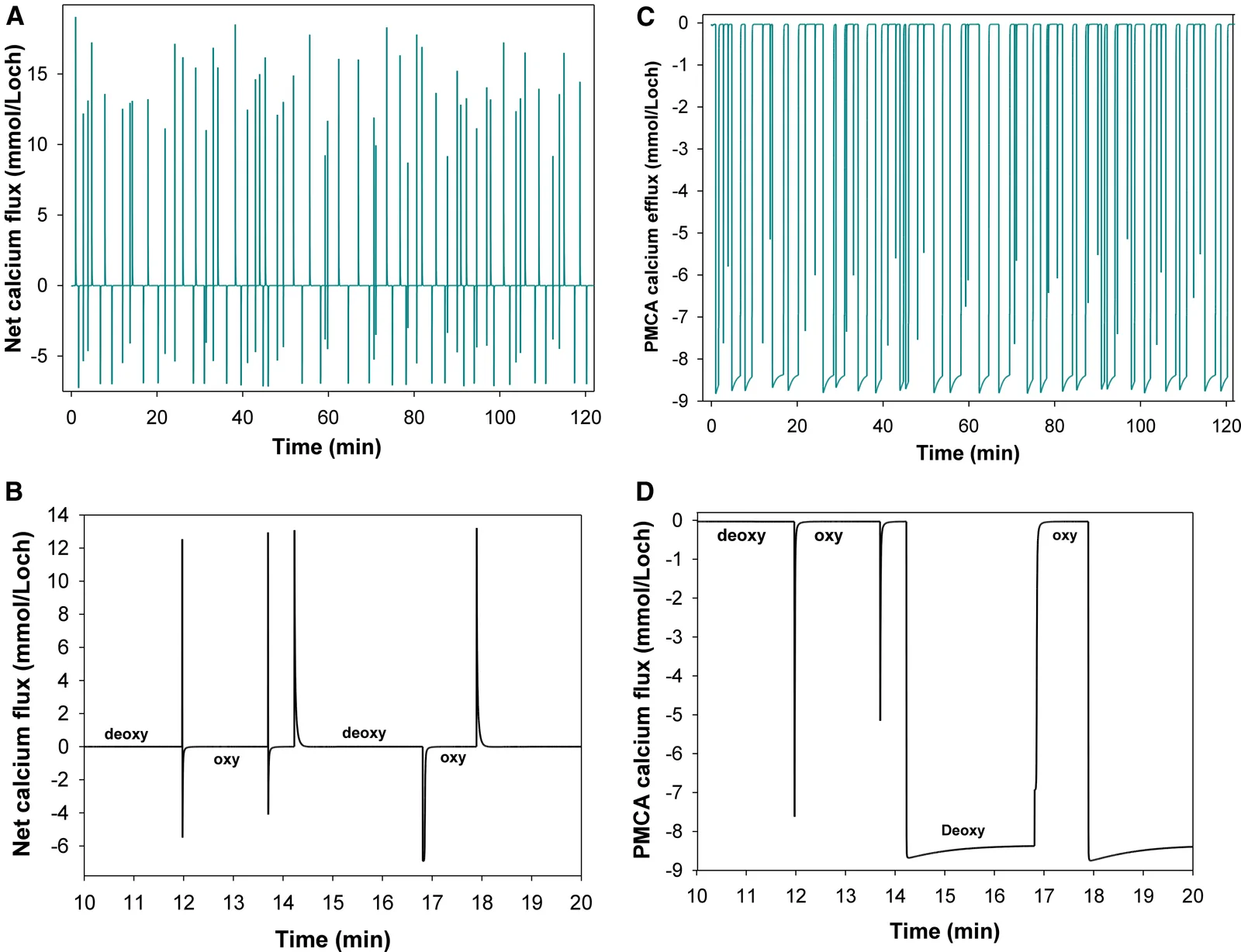
Predicted pattern of net calcium fluxes and calcium pump activity in F-cells during oxy-deoxy intercapillary transits The figure illustrates the effects of alternating pairs of deoxy periods with open and closed Piezo1 channels on net pump-leak calcium fluxes (A and B) and on the kinetics of calcium pump-mediated calcium fluxes (C and D). Comparisons between corresponding panes in Figures 1, 3, and 4 clearly show the intermediate condition of F-cells between normal RBCs (Figure 1) and sickle diskocytes (Figure 3) on the pump-leak calcium kinetics as affected by the reduction in deoxy-Piezo1 activity in the F-cells.

**Figure 5:**
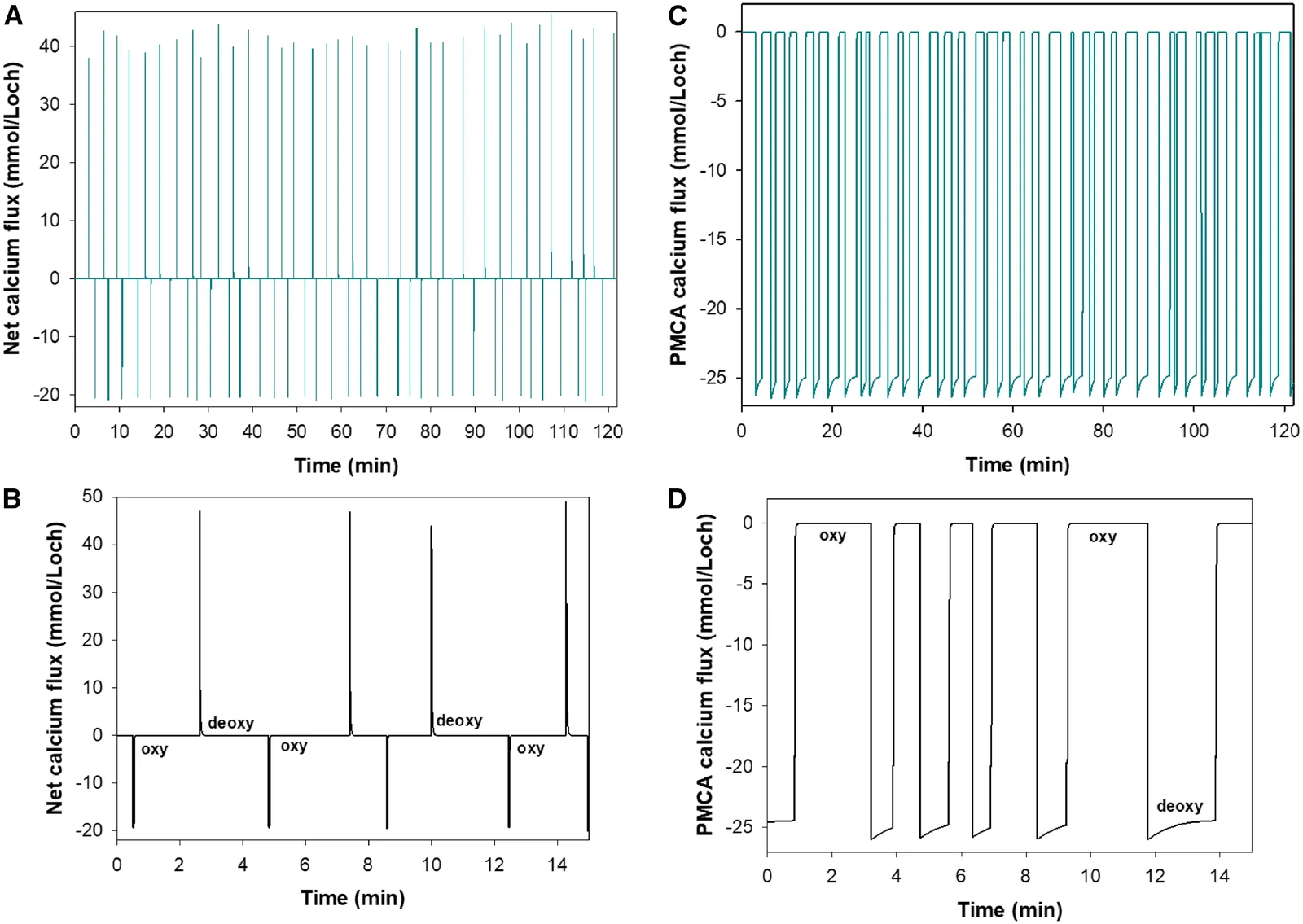
Patterns of Piezo1-induced calcium fluxes in irreversibly sickled cells during capillary transits. (A)–(D) report the same variables as (A)–(D) of Figures 1, 3, and 4 for optimal comparability. The time segments shown were selected from within the hyperdense pathogenic stage of the ISC lifespan.^15^

**Figure 6:**
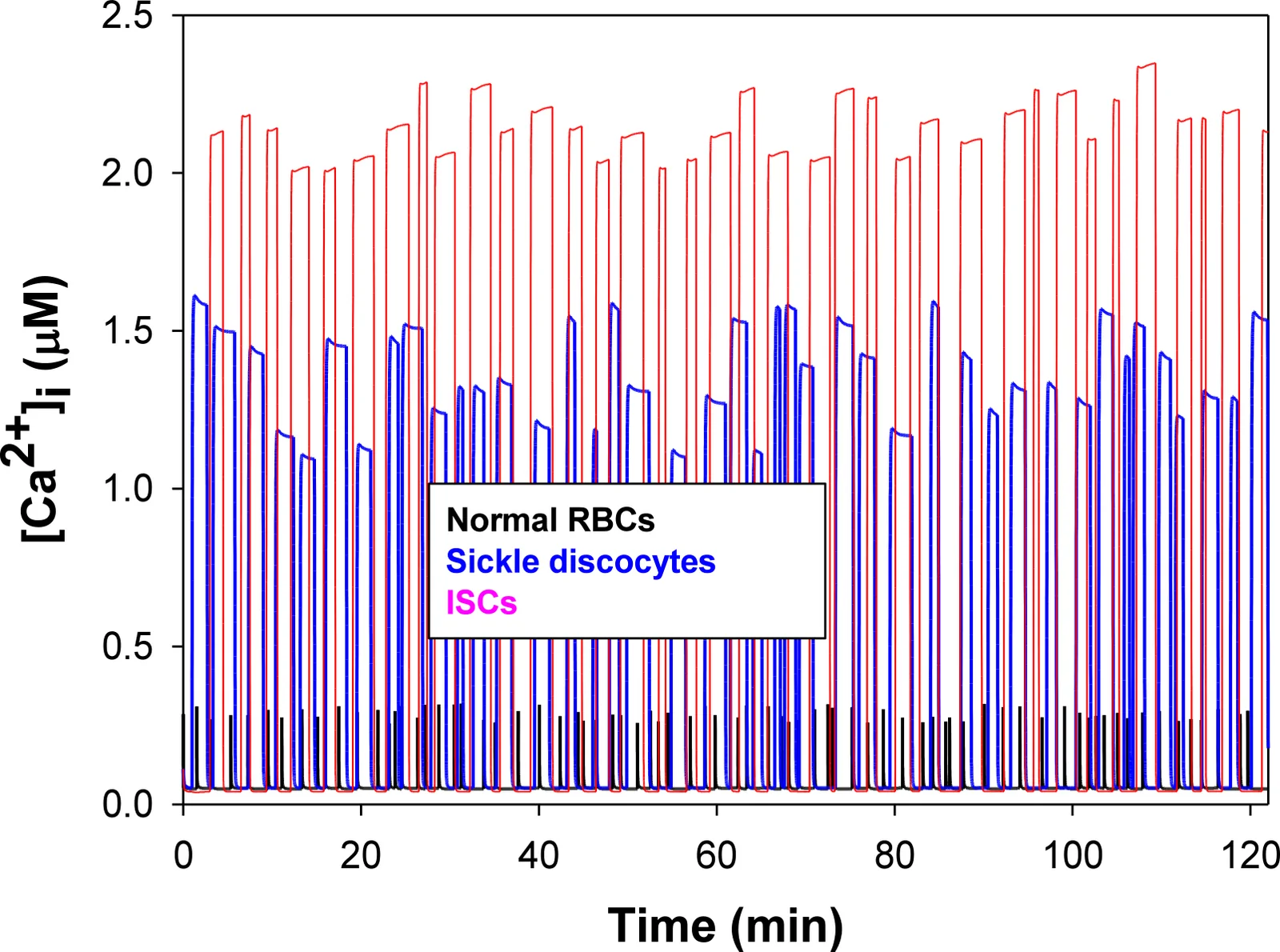
Comparison of [Ca^2+^]_i_ changes in normal red blood cells (black), sickle diskocytes or F-cells (blue) and irreversibly sickled cells (ISCs, red) during successive oxy-deoxy capillary transits in the circulation. The heights and durations of the deoxy [Ca^2+^]_i_ towers and of the oxy intervals vary within the random limits set for the deoxy-Piezo1 permeability and for the duration of intercapillary crossings in the protocols. The calcium flux patterns of F-cells (Figures 4B and 4D) would generate [Ca^2+^]_i_ increases like those of normal RBCs (black) during inactive Piezo1 deoxy periods, and like those of sickle diskocytes (blue) during active deoxy-Piezo1 periods.

The two most relevant properties of Piezo1 channels in the context of this study are the stochastically generated permeability levels simulated for each of the sub second oxy-deoxy RBC transitions,^13^ and, specific to sickle cells, the persistent open state of the channels for the full duration of each deoxy transit.^15^^,^^35^^,^^36^ The downgradient influx of Ca^2+^ and Na^+^ and efflux of K^+^ during Piezo1 openings elevates the cytoplasmic [Ca^2+^] and [Na^+^] concentrations and reduces the [K^+^] concentration stimulating compensatory pump-mediated fluxes. In Figures 1, 3, 4 and 5, the variables chosen to report this highly dynamic process were the net, pump-leak calcium fluxes in panels A and B, and the PMCA-mediated Ca^2+^ extrusion fluxes in panels C and D. Identical display formats were chosen for optimal comparisons between normal RBCs (Figure 1), sickle discocytes (Figure 3), sickle F-cells (Figure 4), and irreversible sickle cells (Figure 5). The dynamics of the [Ca^2+^]_i_ changes in normal RBCs and sickle cell subtypes are shown combined in Figure 6. In Figures 1, 3, 4, and 5, short time-segments were selected in panels B and D to display the results with sufficient time-resolution to appreciate the singularities of single oxy-deoxy transitions; the overall pattern that applies throughout the full lifespan of each cell type is shown in panels A and C. Analysis of the sodium-potassium pump-leak results in ISCs was done on previously published figures and data^15^ as explained in results.

## Results

The predicted Piezo1 effects on the pump-leak calcium fluxes of “normal” RBCs in the circulation are reported first to offer a comparative framework for the characterization of the altered Piezo1 responses in sickle cell subtypes.

### Net calcium fluxes of normal RBCs

Deformation of RBCs on capillary entries, whether to oxy or deoxy streams, activates a variable proportion of the membrane Piezo1 channels for a fraction of a second. This causes a sharp, brief, and poorly selective increase in the ion permeability of the RBC membrane, kindling quantal dissipation steps of all ionic electrochemical gradients, the inward calcium gradient by far the steepest. The ensuing [Ca^2+^]_i_ elevation, the net result of large leak-influx and pump-efflux, occasionally reaches a level sufficient to activate Gardos channels for a brief instant of time, causing a minimal dehydration step.^3^ Figures 1A and 1B, shows the predicted patterns of net pump-leak calcium fluxes resulting from calcium influx through Piezo1 and calcium extrusion through the PMCA, a clear biphasic pattern causing extremely brief in-out displacements from the near-zero net flux baseline filling over 95% of the time between capillary crossings (Figure 1B). Baseline physiological pump-leak fluxes are around 50 μmol/(Loch),^37^ hardly distinguishable from zero on the y-scales of Figures 1C and 1D, and baseline [Ca^2+^]_I_ levels are about 40–60 nM.

The upward peaks in Figures 1A and 1B show the net calcium influx resulting from each subsecond opening of the Piezo1 channels, amplitude variations reflecting the random number of channels opening on sequential capillary crossings. The downward troughs are the undershoot caused by PMCA-mediated extrusion of the calcium remaining in the cells after Piezo1 channel closures. On the timescale of Figure 1A, the in-out fluxes are so brief and contiguous that they appear as single vertical lines in between the random duration periods between intercapillary transits. On the expanded timescale of Figure 1B, the three main properties of the net calcium fluxes appear more clearly: 1) the sequential nature and brevity of the in-out net flux imbalances on capillary entries; 2) the near-zero net calcium baseline flux during intercapillary transits; and 3) the identity of this kinetic pattern regardless of oxy-deoxy or deoxy-reoxy transits.

Figures 1C and 1D, shows the PMCA-mediated calcium extrusion fluxes triggered by the [Ca^2+^]_i_ surges following Piezo1 openings (Figure 1E). The powerful capacity of the PMCA^38^^,^^39^ returns net calcium fluxes to baseline levels within a few seconds after each transit with no difference between oxy or deoxy transitions (Figures 1B and 1D).

Figure 1E shows the predicted [Ca^2+^]_i_ changes caused by the dynamic interactions between Piezo1 and the PMCA pump during and after capillary crossings. The brief initial Piezo1-induced peaks are rapidly returned to baseline [Ca^2+^]_i_ levels by the PMCA. Although peak [Ca^2+^]_i_ levels reach 50-fold higher levels than the 40–50 nM physiological baseline, their brevity only allows the effects on Gardos channels to accumulate and become detectable over weeks and thousands of capillary transits, detection usually documented by slow and gradual cell densification.^2^^,^^40^^,^^41^

**Figure 2:**
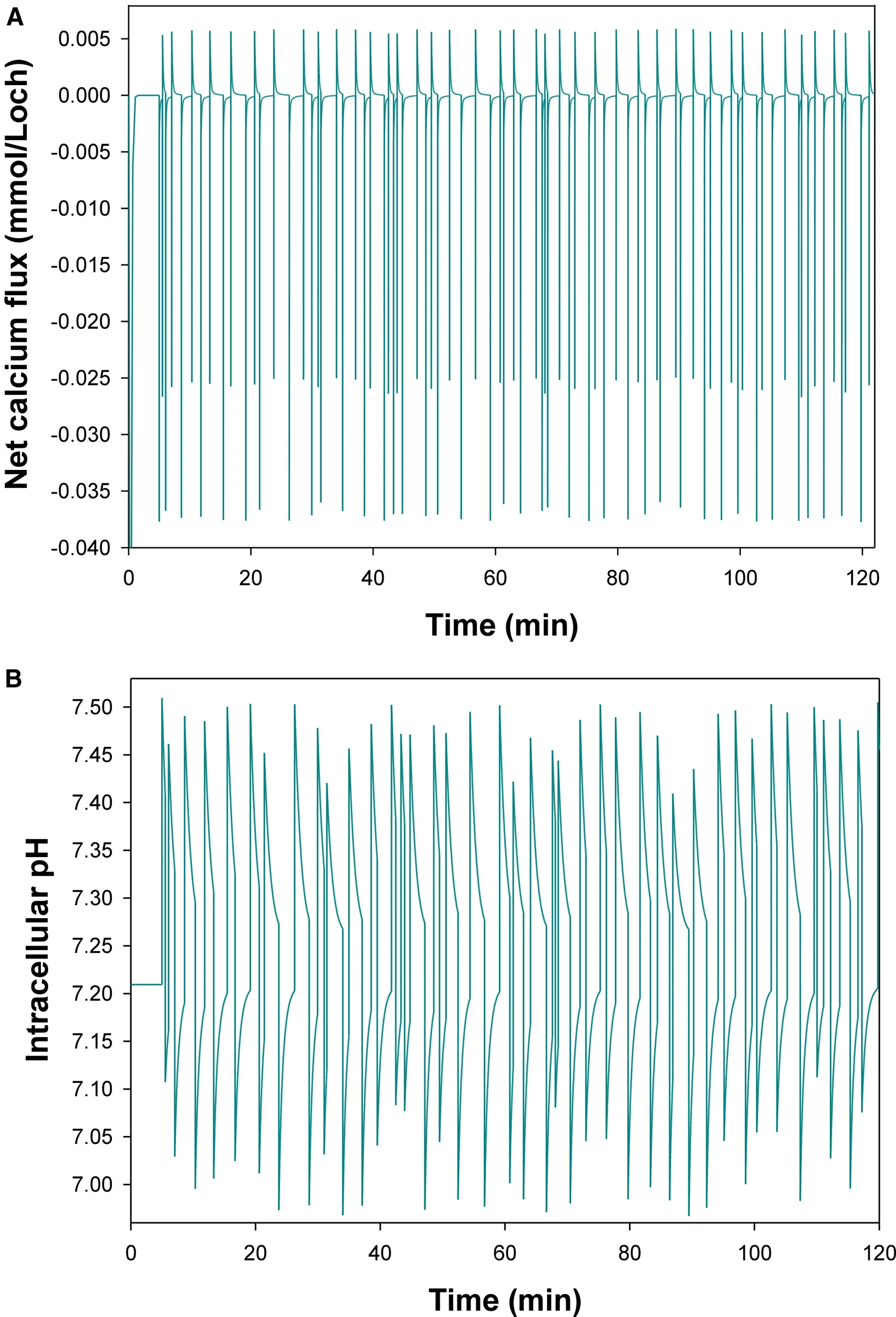
Effects of oxy-deoxy pH changes during capillary crossings on net calcium fluxes in normal red blood cells with Piezo1 channels disabled in the simulations. Oxy-deoxy transitions generate large and opposite intracellular pH shifts in RBCs (B), alkaline shifts on deoxy, acid shifts on reoxy. These pH shifts influence PMCA-mediated fluxes (A) because the pump operates as an electroneutral Ca^2+^:2H^+^ exchanger.^43^ It was therefore important to estimate the potential influence of the oxy-deoxy [H^+^]_i_ shifts on the magnitude and kinetics of the net calcium fluxes, separate from Piezo1 effects, by disabling Piezo1 in the protocol. By comparing the y-axis scales of Figures 2A and 1A, it is clear that the magnitude of the pH effects on net calcium fluxes with Piezo1 disabled are much smaller than those mediated by Piezo1.

Oxy-deoxy transitions induce cell pH changes.^6^^,^^7^^,^^10^^,^^11^^,^^42^ Because the PMCA operates as an electroneutral Ca^2+^:2H^+^ exchanger,^43^ it was important to estimate the relative contributions of Piezo1 and the oxy-deoxy pH changes to the net calcium fluxes triggered by Piezo1. To estimate the pH effects on their own required running the model as for Figure 1 but with Piezo1 disabled. The results in Figure 2B show the large initial pH displacements from a baseline pH of 7.2 to alkaline peaks near 7.7 on oxy-deoxy transits, and to peaks just below 7.0 on deoxy-reoxy transits. The initial pH displacements rapidly converge to much closer and steady deoxy and oxy levels, never attaining steady-state values within most capillary intertransit times of less than 3–4 min. The pH effects on the net calcium fluxes are shown in Figure 2A. The mechanism behind their complex kinetics has been analyzed before.^11^ What matters in the current context is that the magnitudes of the pH effects on net calcium fluxes are between two and three orders of magnitude smaller than those of the Piezo1-induced fluxes shown in Figure 1A, rendering the pH contribution undetectable on the y-axis scale of that figure.

### Net calcium fluxes in mature sickle cell diskocytes

The results in Figure 3 show the patterns predicted for the net pump-leak Ca^2+^ fluxes (Figures 3A and 3B) and PMCA-mediated Ca^2+^ extrusion fluxes (Figures 3C and 3D) triggered by Piezo1 on capillary transits in sickle diskocytes.

The instant sickle diskocytes enter a deoxy stream, PIEZO1 channels open as for normal RBCs but remain open for the duration of the deoxy transit (Figures 3A and 3B), the result of a deoxy-disabled spontaneous inactivation process of the Piezo1 channels.^13^^,^^35^ Sustained calcium influx driven by the steep inward calcium gradient elevates [Ca^2+^]_i_ (Figure 6, blue) and stimulates the PMCA to rapidly extrude calcium toward a zero-net pump-leak flux balance (Figures 3A and 3B). But unlike in normal RBCs, where unidirectional pump-leak calcium fluxes return to near-zero baseline levels (Figure 1D), the unidirectional pump-leak calcium fluxes of sickle diskocytes near the zero-net boundary remain very far from baseline levels for the duration of each deoxy transit (Figure 3D). The continuity of the vertical lines on each side of the zero-net horizontal line in the normal RBCs (Figures 1A and 1B) changes to a horizontally displaced up-down pattern at the zero-net boundary in sickle diskocytes (Figures 3A and 3B), hiding very different unidirectional calcium fluxes between oxy and deoxy transits under the same net-zero balance line.

On entering oxy bloodstreams, Piezo1 channels close normally and remain closed, allowing the PMCA to swiftly extrude any residual calcium gained and to restore [Ca^2+^]_i_ (Figure 6) and net calcium fluxes to baseline levels (Figures 3C and 3D). This dynamic generates completely different patterns for the PMCA-mediated calcium fluxes in sickle diskocytes (Figures 3C and 3D) relative to normal RBCs (Figures 1C and 1D).

### Net calcium fluxes in sickle F-cells

The presence of HbF tetramers in F-cells exerts a powerful inhibitory effect on the polymerization kinetics of HbS.^44^^,^^45^ Reduced polymerization frequency, in turn, also reduces the proportion of deoxy transits with enough HbS polymer to keep Piezo1 channels open. In the context of a study on the homeostasis of F-cells in the circulation, the beneficial effects of HbF in F-cells are best represented by alternating deoxy transits with active and inactive Piezo1 channels, as illustrated in Figure 4. This places F-cells in an intermediate scale between normal RBCs (Figure 1) or sickle trait RBCs with HbAS and sickle diskocytes (Figure 3) on the four scores of Piezo1 activity, pump-leak calcium kinetics, dehydration rate, and effects on the clinical condition of sickle cell disease patients.

### Net calcium fluxes in irreversibly sickled cells

The patterns of Piezo1-induced pump-leak calcium fluxes in ISCs (Figures 5A–5D) are similar to those described for sickle diskocytes (Figure 3). The big difference is in the magnitude of the net calcium fluxes, at least double those in sickle diskocytes (compare y-scales between Figures 5C, 5D, 3C, and 3D), a result of the immature, developmentally arrested condition of ISCs as stress reticulocytes, with constitutive pump activities between 10- and 40-fold higher than in mature normal or sickle diskocytes or F-cells.^46^

The sustained activity of the deoxy-Piezo1 channels in ISCs elevates [Ca^2+^]_i_ (Figure 6) to levels that activate Gardos channels sufficiently to bring the membrane potential closer to the potassium equilibrium potential during deoxy transits. Hyperpolarization increases the driving force for calcium influx in successive deoxy entries, accelerating KCl loss and dehydration in a positive feedback loop toward the full dissipation of the outward potassium gradient causing the hyperdense collapse of the ISCs within a day or so in the circulation. The dissipation of the potassium gradient puts an end to the dehydration capacity of the calcium-Gardos channel circuit, keeping the ISCs in a maximally dehydrated condition for most of their circulatory lifecycle.^15^^,^^33^^,^^36^

### Net sodium fluxes in irreversibly sickled cells

Because intracellular potassium competes with sodium on the Na/K pump,^47^ an important side effect of the inverse changes in intracellular sodium and potassium concentrations during the hyperdense period is a large increase in Na/K pump activity (Figure 3F and 3G, in reference^15^). In line with tracer-flux measurements in stress reticulocytes,^46^ oxy-deoxy pump-leak unidirectional sodium effluxes during the long hyperdense pathogenic period of ISCs can vary widely within the 40–70 mmol/Loch range, well over an order of magnitude above pump-leak sodium fluxes in normal RBCs.

Moreover, although the high calcium pump activity remains restricted to deoxy states in F-cells and diskocytes (Figures 4D and 3D), in ISCs the Na/K pump retains similar activity levels through oxy and deoxy states. This is because whereas PMCA activity responds to large [Ca^2+^]_i_ differentials between oxy and deoxy states throughout the three stages of ISCs lifespan, the much larger mass of intracellular sodium and potassium contents changes minimally over capillary transits. Changes are thus buffered, keeping the intracellular Na^+^ and K^+^ concentrations oscillating within minor margins over oxy-deoxy transitions.^15^^,^^48^

### Comparison of the oxy-deoxy [Ca^2+^]_i_ dynamic in normal and sickle RBCs

Figure 6 compares the [Ca^2+^]_i_ level changes elicited by Piezo1 activity in the circulation for normal RBCs, sickle diskocytes, and ISCs. A single brief, sharp, and low [Ca^2+^]_i_ peak (black) signals the response of normal RBCs initiating capillary entries regardless of oxy or deoxy streams. [Ca^2+^]_i_ swiftly returns to baseline physiological levels in between transits (Figure 1E). In sickle cells, on the other hand, the [Ca^2+^]_i_ dynamics differs markedly in pattern and magnitude between oxy and deoxy transits. Deoxy transits appear as relatively broad vertical towers of elevated [Ca^2+^]_I_, higher for ISCs (Figure 6, red) than for sickle diskocytes or F-cells (Figure 6, blue) reflecting the relative magnitudes attributed to the respective deoxy-Piezo1 permeabilizations in the simulations. Sickle cells return to baseline [Ca^2+^]_i_ levels only during the oxy intervals between deoxy towers.

The deoxy [Ca^2+^]_i_ values in Figures 1E and 6 stay below 2.5 μM levels. Given the highly sigmoid fourth-power [Ca^2+^]_i_ activation kinetics of Gardos channels in human RBCs,^49^^,^^50^ only deoxy-[Ca^2+^]_i_ levels such as those in ISCs can induce significant cumulative dehydration within hours in the circulation. In sickle diskocytes, F-cells, and normal RBCs, cumulative dehydration proceeds much slower. There is also strong but indirect evidence suggesting that even in the high-calcium-accumulating ISCs, [Ca^2+^]_i_ levels and exposure times remain well within the low boundaries shown in Figure 6. The evidence is based on the fact that inosine-monophosphate accumulation, a definitive and irreversible indicator of elevated calcium exposure in RBCs, was never detected in fresh samples of normal and sickle red blood cells.^14^

## Discussion

The results shown here for normal (Figures 1 and 2) and sickle RBCs (Figures 3, 4, and 5) predict patterns of pump-leak calcium fluxes and [Ca^2+^]_i_ changes (Figure 6) elicited by Piezo1 in the circulation in vivo. Figure 6 shows how the persistent open state of deoxy-Piezo1 channels extends the periods of elevated intracellular [Ca^2+^]_i_ levels in all sickle cell subtypes relative to normal RBCs during deoxy transits. These effects are particularly intense in ISCs (Figures 5 and 6) where deoxy-Piezo1 hyperactivity causes the cells’ hyperdense collapse shortly after bone marrow release.

The results of Romero et al.^30^ briefly outlined in the Introduction, suggest that the enhanced ISC response is not simply the result of a higher surface density of channels or of a higher proportion of channels responding to deformation on capillary ingress, but a predisposing result of the altered membrane lipid composition of ISCs affecting channel kinetics, in line with evidence from experimental results in other cell types.^51^^,^^52^^,^^53^^,^^54^^,^^55^ In addition, and only in ISCs, deoxy-Piezo1-mediated ion fluxes were found to be stimulated by heparin and inhibited by high bumetanide concentrations.^33^^,^^36^ The lipid and cytoskeletal membrane environments of stress reticulocytes, the macrocytic ISC precursors,^27^ were shown to differ markedly from those of normally maturing RBCs, and also to be in a very dynamic state of change after bone marrow release.^32^ Taken together, these results point to a multiplicity of factors affecting deoxy-Piezo1 channel function, selectively in ISCs.

In order to explore the effects of the enhanced pump-leak activity of hypoxic sickle cells on the generation of lactic acid in vivo, we start by estimating the overall pump-leak ATP turnover of sickle cells. The current simulations suggest that PMCA-mediated Ca^2+^ flux rates in deoxy ISCs, at about 25 mmol/Loch (Figure 5D), are much higher than those of in sickle diskocytes (Figure 3D) and in F-cells during active deoxy-Piezo1 periods (Figure 4D), at about 8–9 mmol/Loch. As explained in Results (Net sodium fluxes in irreversibly sickled cells), Na/K pump-mediated Na^+^ efflux rates in ISCs are around 40 to 70 mmol/Loch,^15^^,^^46^ both in oxy and deoxy states. The Ca^2+^:ATP stoichiometry of the PMCA is 1:1, whereas the Na^+^:ATP ratio of the sodium pump is 3:1. Therefore, mean ATP consumption by the PMCA in the bloodstream will be about 4.5 mmol/Loch for sickle diskocytes and about 12–13 mmol/Loch for ISCs (time averaged over half deoxy-half oxy periods). For the sodium pump, ATP consumption rates of ISCs in the bloodstream will be around 12–15 mmol/Loch over both oxy and deoxy periods.

The ATP content of density-separated sickle cells from fresh blood samples was shown to be similar or slightly higher than that of normal RBCs for all density fractions.^31^ Therefore, the balance between ATP consumption and metabolic synthesis is sustained throughout the cells’ lifespans for all sickle cell subtypes including reticulocytes, sickle diskocytes, F-cells, and ISCs. Glycolytic metabolism generates one lactic acid molecule per each ATP molecule synthesized. From the pump-leak values in Figures 3, 4, and 5, rough estimates of pump-mediated ATP consumption and lactic acid production rates for sickle diskocytes and F-cells would be around 6 mmol/Loch, and for ISCs and immature reticulated forms, around 50–60 mmol/Loch.

These rates of ATP turnover represent a vastly increased source of relentless lactate production by the large body mass of RBCs, even allowing for the anemic condition of patients. Unlike the transients of exercise-generated lactate, it is the sustained nature of this pump-leak lactate generator operating on overdrive within sickle cells that elevates baseline plasma lactate concentrations^56^ and generates metabolic conditions that render patients with SCD particularly vulnerable to liver or kidney complications affecting lactate metabolism or excretion.

On a per-cell ranking, the current results suggest that the pump-leak lactate generator operates with decreasing intensity in stress reticulocytes, reticulocytes, ISCs, sickle diskocytes, and F-cells. The lactate production burden will thus be determined by the proportion of these cell types in the bloodstream of each patient. Additional factors potentially influencing pump-leak-induced lactatemia in sickle cell disease are the rate-balance between lactate generation, export to plasma, and intra- and extracellular pyruvate conversion by lactic dehydrogenase activity.^25^ These considerations illustrate the multifactorial nature of the potential effects of elevated pump-leak lactate generation within sickle cells, knowledge expected to be of help in the management of metabolic acidosis in sickle cell disease patients.

## Data availability

The simulated data sets and underlying model parameters supporting the results of this study are available for download with open access from a GitHub repository (https://github.com/sdrogers/redcellmodeljava), together with the model code, comprehensive user guides, tutorials, and an updated file with the governing equations of the model. The updated model version used here, RCM 1.0.3.jar, operates as a program within the JAVA environment. Model simulations follow user-generated instructions recorded in editable protocol files (^∗^.txt). The protocol files used for the simulations reported here for each figure are available for downloading within the “Releases” link in the repository (the “Sickle cell pump-leak” set), together with the RCM 1.0.3.jar program. Users can thus easily replicate results, edit protocols, and investigate further.

The protocol files for each of the figures can be directly uploaded on RCM 1.0.3.jar by choosing the “Load from file” tag and clicking on “Run Model.” When the run ends, results are immediately available for inspection in graphic format by clicking “Launch plotter” and by selecting the variables to be inspected from the long list on the left. The variable names used for the figures displayed here are as follows: “FCa” for the “Net calcium flux” ordinates in Figures 1, 3, 4, and 5, panels A and B; “FCaP” for “PMCA calcium efflux” ordinates in Figures 1, 3, 4, and 5, panels C and D; and “CCa2+” for the “[Ca^2+^]_i_” ordinates in Figures 1E and 6. The y-axis in Figure 2B reports cytoplasmic pH, the “pHi” model variable. These same variable names are used to head the columns on the ^∗^.csv files that are generated by pressing the “Save output” tag. Time output, in minutes, is on column 1. Selected columns from ^∗^.csv data files were imported to Sigmaplot to generate the reported figures.

## Acknowledgments

We are grateful to Teresa Tiffert for helpful and insightful comments on the manuscript. This work was supported by funds from The University of Cambridge and from 10.13039/501100000853The University of Glasgow.

## Author contributions

The computational components of this research were discussed and drafted in close collaboration by both authors. The biophysical and medical aspects were dealt with by V.L.L.

## Declaration of interests

The authors declare no competing interests.
